# Detection of high-risk patients resistant to CDK4/6 inhibitors with hormone receptor-positive HER2-negative advanced and metastatic breast cancer in Japan (KBCSG-TR-1316)

**DOI:** 10.1007/s12282-023-01485-y

**Published:** 2023-07-24

**Authors:** Manabu Futamura, Takahiro Nakayama, Tetsuhiro Yoshinami, Chiya Oshiro, Mikiya Ishihara, Midori Morita, Akira Watanabe, Azusa Tanigichi, Masami Tsukabe, Masafumi Shimoda, Kanae Nitta, Yoko Chihara, Hiroyuki Yasojima, Yoshimi Ouchi, Yoshihisa Tokumaru, Norikazu Masuda

**Affiliations:** 1 Department of Breast Surgery, Gifu University Hospital, 1-1 Yanagido, Gifu, 501-1194 Japan; 2 Department of Breast and Endocrine Surgery, Osaka International Cancer Institute, Osaka, Japan; 3 Department of Breast and Endocrine Surgery, Osaka University Graduate School of Medicine, Osaka, Japan; 4 Department of Breast Surgery, Kaizuka City Hospital, Kaizuka, Japan; 5 Mie University Hospital Cancer Center, Tsu, Japan; 6 Division of Endocrine and Breast Surgery, Kyoto Prefectural University of Medicine, Kyoto, Japan; 7 Department of Breast and Endocrine Surgery, Osaka Police Hospital, Osaka, Japan; 8 Breast and Endocrine Surgery, Otemae Hospital, Osaka, Japan; 9 Department of Breast Surgery, Itami City Hospital, Itami, Japan; 10 Department of Surgery Breast Oncology, NHO Osaka National Hospital, Osaka, Japan; 11 Department of Breast Surgery, Saiseikai Shiga Hospital, Ritto, Japan; 12 Department of Breast and Endocrine Surgery, Nagoya University Graduate School of Medicine, Nagoya, Japan

**Keywords:** Luminal, Metastatic breast cancer, CDK4/6 inhibitor, Resistant, NOLUS

## Abstract

**Background:**

Cyclin-dependent kinase 4/6 inhibitors (CDK4/6i) improve the prognosis of hormone receptor-positive HER2-negative advanced/metastatic breast cancer (HR+/HER2− mBC). However, some cancers show resistance to CDK4/6i and have a poor prognosis. The non-luminal disease score (NOLUS) was developed to predict non-luminal disease using immunohistochemical analysis.

**Methods:**

The association between the efficacy of CDK4/6i and NOLUS was investigated by evaluating pathological and clinical data, including real-world progression-free survival (rw-PFS) and overall survival (OS). Real-world data of patients with HR+/HER2− mBC who received CDK4/6i therapy [palbociclib or abemaciclib] as first- or second-line endocrine treatments was obtained. NOLUS was calculated using the formula: NOLUS (0–100) = − 0.45 × estrogen receptor (ER) (%) − 0.28 × progesterone receptor (PR) (%) + 0.27 × Ki67(%) + 73, and the patients were divided into two groups: NOLUS-positive (≥ 51.38) and NOLUS-negative (< 51.38).

**Results:**

Of the 300 patients, 28 (9.3%) were NOLUS-positive, and 272 (90.7%) were NOLUS-negative. The expression rates (%) of ER and PgR in NOLUS-positive patients were lower than those in NOLUS-negative patients (*p* < 0.001). Ki67 expression was higher in NOLUS-positive patients. There were statistically significant differences in prognosis (rw-PFS and OS) between the two groups. Moreover, NOLUS-negative patients showed statistically better rw-PFS with first-line therapy than second-line therapy. However, NOLUS-positive patients showed poor prognoses with both the first and second therapeutic lines, suggesting CDK4/6i inefficacy for NOLUS-positive patients.

**Conclusions:**

The efficacy and prognosis of CDK4/6i significantly differed between the NOLUS-positive and NOLUS-negative patients. This feasible method can predict patients with HR+/HER2− mBC resistant to CDK4/6i and help select a better therapeutic approach to overcome resistance.

**Supplementary Information:**

The online version contains supplementary material available at 10.1007/s12282-023-01485-y.

## Introduction

Approximately 70% of patients with breast cancer (BC) worldwide belong to the luminal subtype, including the hormone receptor (HR) [estrogen receptor (ER) and/or progesterone receptor (PgR)]-positive (HR+) and human epidermal growth factor (HER2)-negative (HER2−) subtypes [1, 2]. Although the development of adjuvant endocrine therapies (ET) in patients with early-stage BC has reduced the relapse rate, some patients relapse even during or after the completion of adjuvant ET. Single-agent therapy with tamoxifen or aromatase inhibitors (AI) is widely used for an adjuvant ET. During the no-CDK4/6i era, fulvestrant (FUL), a selective estrogen receptor degrader, was used as a second-line ET in patients with HR+HER2− metastatic breast cancer (mBC), de novo stage 4 patients without visceral metastasis, or patients who relapsed during or immediately after adjuvant TAM/AI. However, more effective therapies are needed to overcome resistance to ET [3].

Cyclin-dependent kinases (CDKs) play an important role in regulating the cell cycle. CDK4 and CDK6 interact with cyclin-D and promote hyperphosphorylation of retinoblastoma (Rb) protein, leading to the progression of the cell cycle from the G1 phase to the S phase. Dysregulation of this cell cycle has been reported in many cancers, including breast cancer. In recent years, several small molecules such as palbociclib (PAL) (Ibrance^®^, Pfizer), abemaciclib (ABE) (Verzenio^®^, Eli Lilly), and ribociclib (RIB) (Kisqali^®^, Novartis) have been developed [4]. These CDK4/6 inhibitors (CDK4/6is) have been shown to inhibit tumor progression, resulting in a better prognosis in combination with ET agents such as AI and FUL. The progression-free survivals (PFSs) of first- or second-line therapies using an ET agent with a CDK 4/6i have been shown to significantly improve [5–8]. Adding CDK4/6i to ET can prolong the overall survival in HR+/HER2− mBC [9, 10]. Consistent with the results in the overall population, both PAL and ABE plus AI or FUL have also been shown to be effective in the Japanese subpopulation [11–14]. Indeed, CDK4/6is are effective and key drugs for HR+/HER2− mBC. Moreover, various resistance mechanisms have been identified, such as alterations of the cyclin-D-CDK4/6-Rb pathway and genetic clonal evolution by driver mutations, such as phosphatidylinositol-4,5-bisphosphate 3-kinase catalytic subunit alpha (PIK3CA) and estrogen receptor 1 (ESR1), or cyclin E1 overexpression [15, 16]. According to data from PALOMA 2/3 and MONARCH 2/3, approximately 20% of patients progress within 6 months from commencing CDK4/6is [5–8]. A clinically critical point is how to predict sensitivity or resistance before initiating CDK4/6is to avoid ineffective treatments.

One of the most functional gene profiling tests is the prediction analysis of microarrays using the 50-gene classifier (PAM50), which was developed using quantitative reverse transcriptase polymerase chain reaction or microarray to predict gene expression-based intrinsic subtypes and chemotherapy benefits [17, 18]. Despite the clinical significance of predictive and prognostic information for BC, this test has not been widely used because of its high cost and sample preparation. Recently, Pascual et al. reported a unique pathology-based method called the non-luminal disease score (NOLUS) that can compensate for these disadvantages. NOLUS was calculated using the expression of ER, PgR, and Ki67 by immunohistochemistry (IHC), in which non-luminal tumors were defined as NOLUS-positive (≥ 51.38) and luminal tumors as NOLUS-negative (< 51.38) [19]. Further studies demonstrated that patients with basal and HER2 subtypes in the NOLUS-positive population showed poorer prognoses than those in the NOLUS-negative population [20]. These findings prompted us to characterize NOLUS in Japanese patients who received CDK4/6is. Since 2017, two CDK4/6is, PAL and ABE, have been approved and are available for mBC. In this study, we retrospectively investigated the association between NOLUS and clinicopathological data from the patients treated with CDK4/6is in Japan, sponsored by the Kinki Breast Cancer Study Group-Translational Research (KBCSG-TR) to investigate the usefulness of NOLUS.

## Materials and methods

### Search strategy and study design

This multicenter retrospective observational study was approved by the Central Ethics Committee of Gifu University. Between December 2017 and December 2021, real-world data of patients with HR+/HER2− mBC who received CDK4/6is were collected from 11 institutes in Japan. Clinicopathological data were obtained from patients who received CDK4/6is, such as PAL or ABE, as first- or second-line ET. The association between NOLUS and prognostic values was analyzed in these Japanese patients. This study was registered at the UMIN-CTR (no. 000049957).

### Eligibility criteria

Pathological data, including the expression levels of ER, PgR, HER2, and Ki67, were evaluated by experienced pathologists in each institute using formalin-embedded tumor samples from either primary or metastatic tumors (metastatic tumors were recommended as long as possible). ER and PgR positivity was defined as > 1% positive tumor cells according to the ASCO/CAP guidelines [21]. In the case of evaluation using the Allred score, the samples were re-evaluated based on ASCO/CAP guidelines [22]. The Ki67 value was quantified using the 2011 Guidelines published by the International Ki67 in the Breast Cancer Working Group [23]. HER2 expression was evaluated according to the ASCO/CAP guidelines 2018 [24]. The clinical data of the patients were obtained from each institute.

### NOLUS calculation

NOLUS was reported by Pascual et al. [19] and was calculated using the following formula: NOLUS (0–100) = − 0.45 × ER(%) − 0.28 × PR(%) + 0.27 × Ki67(%) + 73. The patients were divided into two groups: NOLUS-positive (non-luminal disease) patients with a NOLUS score ≥ 51.38 and NOLUS-negative (luminal disease) patients with a NOLUS score < 51.38.

### Primary outcomes

The primary outcomes of this study were real-world progression-free survival (rw-PFS) and overall survival (OS) [25]. rw-PFS was defined as the period from (1) the starting date of combination therapy to progressive disease (PD). ET and CDK4/6i were started and stopped simultaneously; (2) the starting date of combination therapy to PD when CDK4/6i was interrupted due to adverse events or patients' preference. ET was continued to PD even if CDK4/6i was stopped earlier than ET; and (3) the starting date of endocrine monotherapy to PD when CDK4/6i was added to endocrine monotherapy (before PD). OS was defined as the time from the beginning of the therapy (1–3) to death from any causes.

### Data collection and statistical analysis

Real-world clinical data associated with CDK4/6i was obtained from patient medical records of each institute. Patient characteristics are described as medians and interquartile ranges (IQRs) for continuous variables and frequencies for categorical variables. Ages between groups were compared by the Wilcoxon rank sum test. The expression levels of ER, PgR, HER2, and Ki67 in each group were compared using Fisher's exact tests. The prognosis, including rw-PFS and OS rates, was estimated using the Kaplan–Meier method. The rw-PFS and OS rates between groups were compared using the log-rank test. The hazard ratios and 95% confidence intervals (CIs) were calculated using the Cox proportional hazards regression model. Two-sided p < 0.05 was considered statistically significant. All statistical analyses were performed using R software version 4.2.2 (www.r-project.org).

## Results

### Clinical features of NOLUS-positive/negative patients

**Fig. 1:**
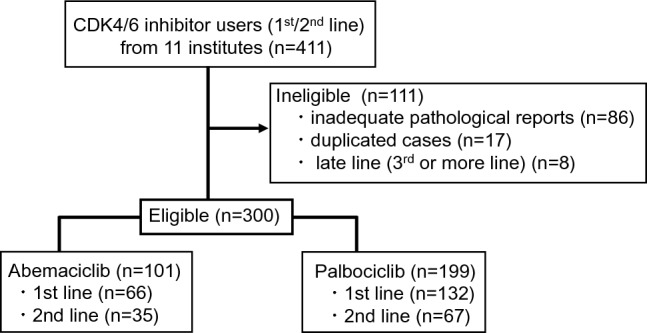
CONSORT diagram. Inclusion/exclusion criteria for patients are shown

Real-world clinical data on the 411 patients who received CDK4/6i as the first- or second-line endocrine therapy from 11 institutes were collected. Of these patients, 300 cases were eligible (adequate group), and 111 were ineligible (86, inadequate pathological reports; 17, duplicated cases; 8, third-line or more ETs received) (Fig. 1). Initially, we evaluated the quality of the adequate group because we were worried there might be significant differences between the two groups. Of the 86 patients with inadequate data, the duration of combination therapy and prognosis were evaluable in 84 patients (inadequate group). The prognostic data between the adequate and inadequate groups were compared. rw-PFS was poorer in the adequate group than in the inadequate group, suggesting that the adequate group includes patients with poorer prognoses. However, no apparent difference was found in OS and the characteristics of patients between the adequate and inadequate groups (Suppl. Figure 1, Suppl. Table 1); thus, we decided to calculate the NOLUS and investigated the clinical significance using the adequate group. Of the 300 patients, 28 were NOLUS-positive (9.3%), and 272 were NOLUS-negative (90.7%). IHC was evaluated using primary (*n* = 254, 84.7%) or metastatic (*n* = 46, 15.3%) tumors. A total of 198 (66%) received CDK4/6is as first-line therapy and 102 (24%) received it as second-line therapy. As two CDK4/6is are available in Japan, PAL (*n* = 199, 66.3%) and ABE (*n* = 101, 33.7%) were used. Combination endocrine therapies included aromatase inhibitors [*n* = 112 (37.3%)] (letrozole: 88, anastrozole: 23, exemestane: 1), fulvestrant [± luteinizing hormone-releasing hormone agonist (LH-RH-ag)] [*n* = 180 (60%)], and tamoxifen + LH-RH agonist [*n* = 1 (0.4%)] (Table 1).

**Table 1:** Patient characteristics

	NOLUS-positive	NOLUS-negative	*p* value
Number of cases	28	272	
Age (median)	61.5	61	0.454
IHC evaluation for NOLUS			
Primary	21 (75%)	233 (85.7%)	0.165
Metastatic	7 (25%)	39 (14.3%)	
Therapy line			
1st line	19 (67.9%)	179 (65.8%)	> 0.999
2nd line	9 (32.1%)	93 (34.2%)	
CDK4/6 inhibitor			
PAL	17 (60.7%)	182 (66.9%)	0.532
ABE	11 (39.3%)	90 (33.1%)	
Combinational endocrine therapy			
LET	3 (10.7%)	85 (31.2%)	0.009
ANA	4 (14.3%)	19 (7.0%)	
EXE	1 (3.6%)	0 (0.0%)	
FUL	18 (64.3%)	156 (57.4%)	
FUL + LH-RH ag	2 (7.1%)	4 (1.5%)	
TAM + LH-RH ag	0(0.0%)	1 (0.4%)	
HER2			0.086
0	12 (42.9%)	80 (30.4%)	
1	8 (28.6%)	136 (51.7%)	
2	8 (28.6%)	47 (17.9%)	
3	0 (0.0%)	1 (0.4%)	

Statistical analysis was by either Wilcoxon rank sum test or Fisher's exact test

*IHC* immunohistochemistry, *NOLUS* non-luminal disease score, *PAL* palbociclib, *ABE* abemaciclib, *LET* letrozole, *ANA* anastrozole, *EXE* exemestane, *FUL* fulvestrant, *LH-RH ag* luteinizing hormone-releasing hormone agonist, *TAM* tamoxifen

### Expression rates of IHC in NOLUS-positive/negative groups

**Fig. 2:**
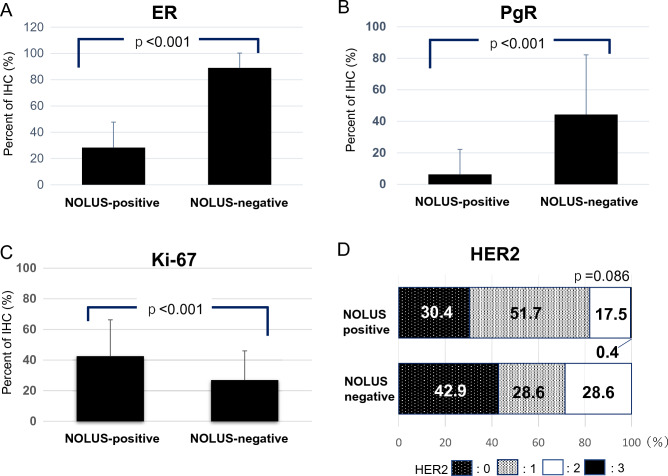
Expression rates (%) of biomarkers in NOLUS-positive (*n* = 28) and NOLUS-negative (*n* = 272) patients. **A** Estrogen receptor (ER), **B** progesterone receptor (PgR), **C** Ki67, and **D** human epidermal growth factor (HER2) were indicated, respectively, by mean ± SD

The expression rates (%) in NOLUS-positive (*n* = 28) and NOLUS-negative (*n* = 272) patients were 28.2 ± 19.4 and 89.0 ± 11.3 for ER (*p* < 0.001), 6.3 ± 15.9 and 44.3 ± 37.9 for PgR (*p* < 0.001), and 42.5 ± 23.8 and 26.9 ± 19.1 for Ki67 (*p* < 0.001), respectively. HER2 expression (score 0, 1, 2 and ISH-negative, 3) was 42.9%, 28.6%, 28.6%, and 0% for NOLUS-positive and 30.4%, 61.7%, 17.5%, and 0.4% for NOLUS-negative (*p* = 0.086), respectively (Fig. 2). There were statistical differences in the expression rates of ER, PgR, and Ki67 between the NOLUS-positive and NOLUS-negative groups.

### Association between NOLUS and prognosis

**Fig. 3:**
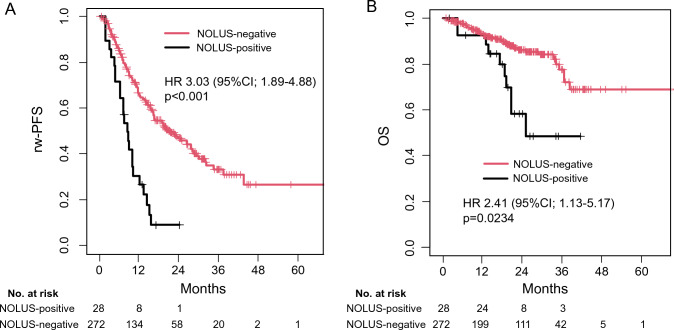
Kaplan–Meier curve estimates between NOLUS-positive and NOLUS-negative patients by all patients. **A** Real-world progression-free survival (rw-PFS) and **B** overall survival (OS) were demonstrated

The prognostic values between NOLUS-positive and NOLUS-negative patients were compared. The median rw-PFSs were 8.6 months for NOLUS-positive patients and 21.5 months for NOLUS-negative patients. The 6-month and 1-year rw-PFSs were 71.4% and 30.5% for NOLUS-positive patients and 85.2% and 66.6% for NOLUS-negative patients, respectively (HR: 3.03; 95% CI 1.89–4.88, *p* < 0.001) (Fig. 3A). The median OSs were 27.3 months for NOLUS-positive patients and 102.3 months for NOLUS-negative patients. The 6-month and 1-year OSs were 92.6% and 92.6% for NOLUS-positive patients and 97.7% and 93.8% for NOLUS-negative patients, respectively (HR: 2.41; 95% CI 1.13–5.17, *p* = 0.0234) (Fig. 3B). These suggested that NOLUS-positive patients were associated with poor prognoses.

**Fig. 4:**
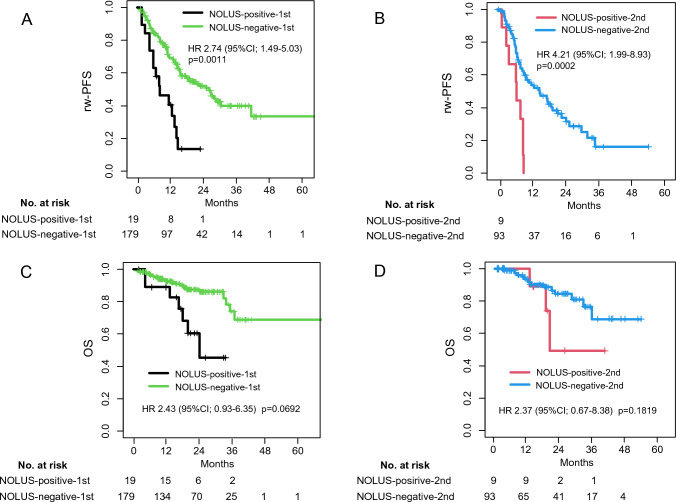
Kaplan–Meier curve estimates subdivided by NOLUS and therapy line. Real-world progression-free survivals (rw-PFSs) for patients treated with first-line therapy (**A**) and second-line therapy (**B**). Overall survivals (OSs) for patients treated with first-line therapy (**C**) and second-line therapy (**D**) were demonstrated, respectively

The prognostic values were further analyzed by classifying them into four groups (NOLUS-positive-first- or NOLUS-positive-second-line therapy and NOLUS-negative-first- or NOLUS-negative-second-line therapy). In patients who received first-line therapy, rw-PFS was 9.1 months for NOLUS-positive and 28.5 months for NOLUS-negative patients. The 6-month and 1-year rw-PFSs were 73.7% and 46.3% for NOLUS-positive patients and 85.4% and 71.3% for NOLUS-negative patients in the first-line therapy (HR: 2.74; 95% CI 1.49–5.03; *p* = 0.0011), respectively (Fig. 4A). In patients who received second-line therapy, rw-PFS was 7.8 months for NOLUS-positive and 17 months days for NOLUS-negative patients. The 6-month and 1-year rw-PFSs were 66.7% and 33.3% for NOLUS-positive patients and 84.8% and 63.9% for NOLUS-negative patients in the second-line therapy (HR: 4.21; 95% CI 1.99–8.93; *p* < 0.001) (Fig. 4B). In patients who received first-line therapy, OS was 27.3 months for NOLUS-positive and 102.3 months for NOLUS-negative patients. The 6-month and 1-year OSs were 88.9% and 88.9% for NOLUS-positive patients and 97.2% and 94.0% for NOLUS-negative patients, respectively (HR: 2.43; 95% CI 10.93–6.35; *p* = 0.0692) (Fig. 4C). In patients who received second-line therapy, OS was 22.4 months for NOLUS-positive and > 100 months (not reached) for NOLUS-negative patients. The 6-month and 1-year OSs were 100% and 100% for NOLUS-positive patients and 98.9% and 93.4% for NOLUS-negative patients (HR: 2.37; 95% CI 0.67–8.38; *p* = 0.1819) (Fig. 4D).

There was no statistical association between HER2 expression and NOLUS (Suppl. Fig. 2).

## Discussion

In this study, 300 patients with HR+/HER2− advanced and metastatic BC who were treated with CDK4/6is were analyzed. NOLUS-positive patients were found to demonstrate poorer prognoses than NOLUS-negative patients. This method is easily calculated using the pathological IHC data and can identify either luminal or non-luminal subtypes. It may also enable us to predict which patients will be resistant to CDK4/6is.

According to the SEER database, the median OS in patients with HR+/HER2− mBC was at most 36 months before the development of CDK4/6is [26]. However, its OS can easily reach more than 40 months because even survival data of second-line therapies from PALOMA-3 and MONARCH-2 resulted in 39.7 and 46.7 months, respectively [9, 10]. In addition, real-world data on second-line therapy using a CDK4/6i also revealed 35.7 months [27]. In the Japanese population, the survival data, including the PFS of PALOMA2/PALOMA3 or MONARCH3/MONARCH2, were almost similar to those of the overall population, in which the declining pattern of each Kaplan–Meier curve revealed a close pattern. This pattern suggests that the combination of CDK4/6i and ET is a promising treatment even for the Japanese population [11–14].

In first-line therapies using PALOMA-2 or MONARCH-3, the addition of CDK4/6i improved the PFS by approximately 15% at 6 months [5, 7]. In addition, in second-line therapies using PALOMA-3 or MONARCH-2, nearly 30% of PFS at 6 months improved [6, 8]. These results indicate that the development of CDK4/6is resulted in a significant survival impact on HR+/HER2− mBC. In contrast, approximately 20% of these patients in first-line therapy progressed within 12 months. Moreover, approximately 30% of those in second-line therapy progressed within 6 months despite the outstanding efficacy of CDK4/6is, indicating that these patients had cancers resistant to CDK4/6is, resulting in poor prognoses.

The mechanisms underlying resistance to CDK4/6is have been investigated in many clinical and basic studies [28]. Impairments in cell cycle regulation and genetic driver mutations such as RB1, PIK3CA, and ESR1 (in particular, Y537S) emerged during treatment with PAL and FUL [15]. Mutated ESR1 is associated with decreased ER dependency, leading to resistance to CDK 4/6is [29]. According to biomarker analysis of the MONALEESA phase III studies, two intrinsic subtypes, HER2-enriched (HER2E) and basal-like, revealed shorter PFS, with subtypes of approximately 15% (HER2E; 10–15%, basal; 2–3%) of all populations. Although HER2E cells responded to RIB, basal-like cells showed almost no response [20]. Therefore, it should be noted that some populations have cancers resistant to CDK4/6is. How we can evaluate the efficacy of CDK4/6is and predict resistance early is critical.

Although PAM50 has been useful for identifying intrinsic subtypes, a substitute is needed because it is not available for daily practice in Japan. In this study, the usefulness of NOLUS at a daily clinical level was investigated. The advantage is that it can be easily calculated only from IHC results without gene expression data, leading to the identification of either non-luminal or luminal disease. Despite a total of 411 patients who received CDK4/6i as first- or second-line therapies, only 300 were analyzed because of incomplete IHC data. Of all the patients examined, 28 (9.3%) were classified as having a non-luminal disease. It is difficult to further separate these into HER2E and basal-type subtypes because there is no detailed method to discriminate non-luminal disease defined by NOLUS ≥ 51.38. However, Pascual et al. demonstrated that more than 90% of non-luminal patients with very high NOLUS ≥ 75 were basal-like and were suggested to have cancers possibly resistant to CDK4/6i. In our study, ten patients with NOLUS ≥ 75 exhibited ER, PgR, and Ki67 positivity of 12.6%, 2.6%, and 56.5%, respectively. In contrast, 18 patients with 51 < NOLUS ≤ 74 exhibited 36.9%, 9.3%, and 33.7%, respectively. Thus, a NOLUS ≥ 75 would reveal possible basal-like features.

The clinical significance of CDK4/6is is clear. In Japan, two types of CDK4/6is have been available since December 2017. According to the Japanese Breast Cancer Society Clinical Practice Guidelines for Breast Cancer 2022, they are currently recommended as the first choice of treatment for HR+/HER2− mBC [30]. Our data demonstrate that NOLUS is useful for Japanese patients with HR+/HER2− mBC to classify the intrinsic subtype and predict the efficacy of CDK4/6is at the daily clinical level. This formula may enable the development of other appropriate treatments. For NOLUS-positive patients with decreased ER dependency, other strategies using different molecular-targeted or chemotherapeutic drugs should be considered [29]. Since at least 198 patients (68.1%) had low HER2 (HER2; 1 or 2) in this study population, chemotherapy or trastuzumab deruxtecan may be a treatment option for patients, particularly those with very high NOLUS ≥ 75, instead of CDK4/6is [31]. Thus, NOLUS may easily aid in the selection of treatments for patients with HR+/HER2− mBC without gene expression data.

This study had some limitations. First, it was a retrospective observational study based on daily clinical data. Second, the sample size was small for the following two reasons. Patient data were collected between December 2017 and December 2021. During this study period, CDK4/6is had been approved and was mainly used not for early-line treatment but for late treatment. Additionally, one-fourth of the patients registered in this study were excluded because of incomplete IHC data, resulting in a lack of NOLUS data. Third, to evaluate NOLUS, we wanted to obtain as much IHC data as possible from metastatic sites. However, only 46 (15.6%) patients were evaluated for metastatic sites. Due to the difficulty of re-biopsy of metastatic sites, we had to use primary tumors (surgically dissected specimens or biopsy samples) instead of metastatic sites. As NOLUS may differ between primary and metastatic sites, these changes may influence the effects of CDK4/6is and its prognosis. Of the 26 patients whose NOLUS was calculated using both primary and metastatic sites, three changed from luminal to non-luminal disease. Fourth, the criteria for IHC evaluation by pathologists may differ in each institute. Some IHC samples evaluated using the Allred score needed to be re-evaluated using the ASCO/CAP guidelines, which were only analyzed using the proportion score [21].

In conclusion, NOLUS could predict the prognosis of patients treated with CDK4/6is. This formula offers an option for selecting an appropriate treatment for patients with HR+/HER2− mBC. Further studies are required to understand the usefulness and reliability of this method as compared to gene expression data.

### Supplementary Information

Below is the link to the electronic supplementary material.

Supplementary file1 Supplementary Fig. 1 Comparison of Kaplan-Meier curve estimates between adequate (n=300) and inadequate (n=84) groups. A) Real-world progression-free survival (rw-PFS) and B) overall survival (OS) were demonstrated, respectively. Supplementary Fig. 2 Kaplan–Meier curve estimates subdivided by NOLUS and human epidermal growth factor (HER2) status. Real-world progression-free survivals (rw-PFSs) of patients in the NOLUS-positive group. rw-PFSs were compared between patients with HER2: 0 and those with HER2: 1,2 (A) and those of patients in the NOLUS-negative group (B). Overall survivals (OSs) of patients in the NOLUS-positive group. Differences in OSs between patients with HER2: 0 and HER2: 1,2,3 (C), and those in the NOLUS-negative group (D) were demonstrated, respectively (PPTX 165 KB)

Supplementary file2 Suppl. Table 1 Patient characteristics of the adequate and inadequate groups. In the inadequate group, some patient medical records containing pathological data were missing because Ki67 was not evaluated in those days or their transition to another hospital without detailed pathological information. Comparisons between groups were performed using Fisher's exact test. PAL, Palbociclib; ABE, Abemaciclib; LET, Letrozole; ANA, Anastrozole; EXE, Exemestane; FUL, Fulvestrant; LH-RH ag, Luteinizing hormone-releasing hormone agonist; TAM, Tamoxifen; NA, Not applicable (XLSX 11 KB)
