# Supplementary material for: Detection of high-risk patients resistant to CDK4/6 inhibitors with hormone receptor-positive HER2-negative advanced and metastatic breast cancer in Japan (KBCSG-TR-1316)
Source: Breast Cancer. 2023 Jul 24;30(6):943–51. doi: 10.1007/s12282-023-01485-y (PMC10587336; doi:10.1007/s12282-023-01485-y)
Supplement: Supplementary file 1 — Supplementary file1 Supplementary Fig. 1 Comparison of Kaplan-Meier curve estimates between adequate (n=300) and inadequate (n=84) groups. A) Real-world progression-free survival (rw-PFS) and B) overall survival (OS) were demonstrated, respectively. Supplementary Fig. 2 Kaplan–Meier curve estimates subdivided by NOLUS and human epidermal growth factor (HER2) status. Real-world progression-free survivals (rw-PFSs) of patients in the NOLUS-positive group. rw-PFSs were compared between patients with HER2: 0 and those with HER2: 1,2 (A) and those of patients in the NOLUS-negative group (B). Overall survivals (OSs) of patients in the NOLUS-positive group. Differences in OSs between patients with HER2: 0 and HER2: 1,2,3 (C), and those in the NOLUS-negative group (D) were demonstrated, respectively (PPTX 165 KB) [file 12282_2023_1485_MOESM1_ESM.pptx]

## Slide 1
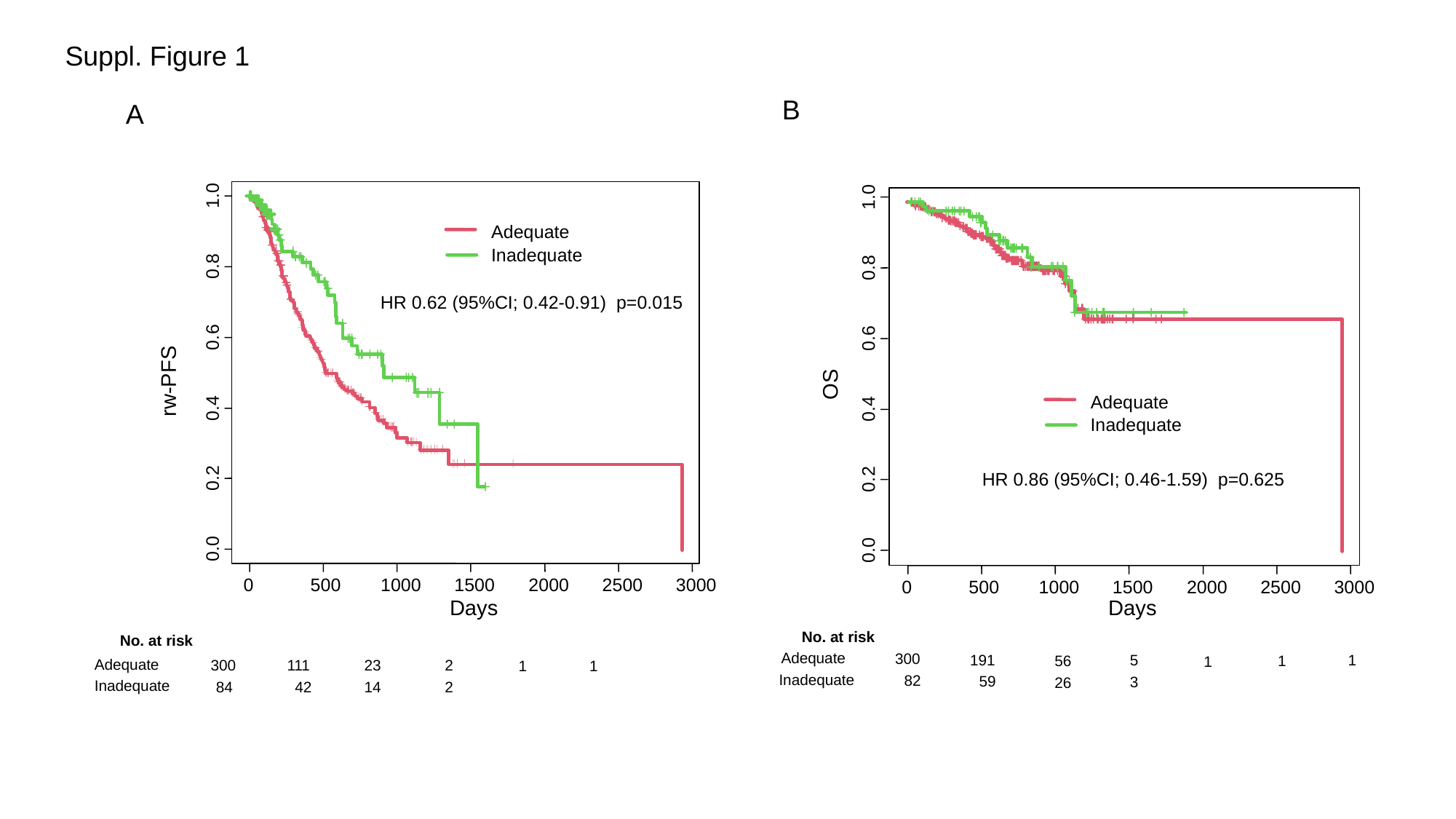

Suppl. Figure 1
B
A
1.0
Adequate
Inadequate
0.8
HR 0.62 (95%CI; 0.42-0.91) p=0.015
0.6
rw-PFS
0.4
0.2
0.0
0
500
1000
1500
2000
2500
3000
Days
No. at risk
Adequate
300
111
23
2
1
1
Inadequate
84
42
14
2
1.0
0.8
0.6
OS
Adequate
Inadequate
0.4
HR 0.86 (95%CI; 0.46-1.59) p=0.625
0.2
0.0
0
500
1000
1500
2000
2500
3000
Days
No. at risk
Adequate
300
191
1
5
56
1
1
Inadequate
82
59
3
26

## Slide 2
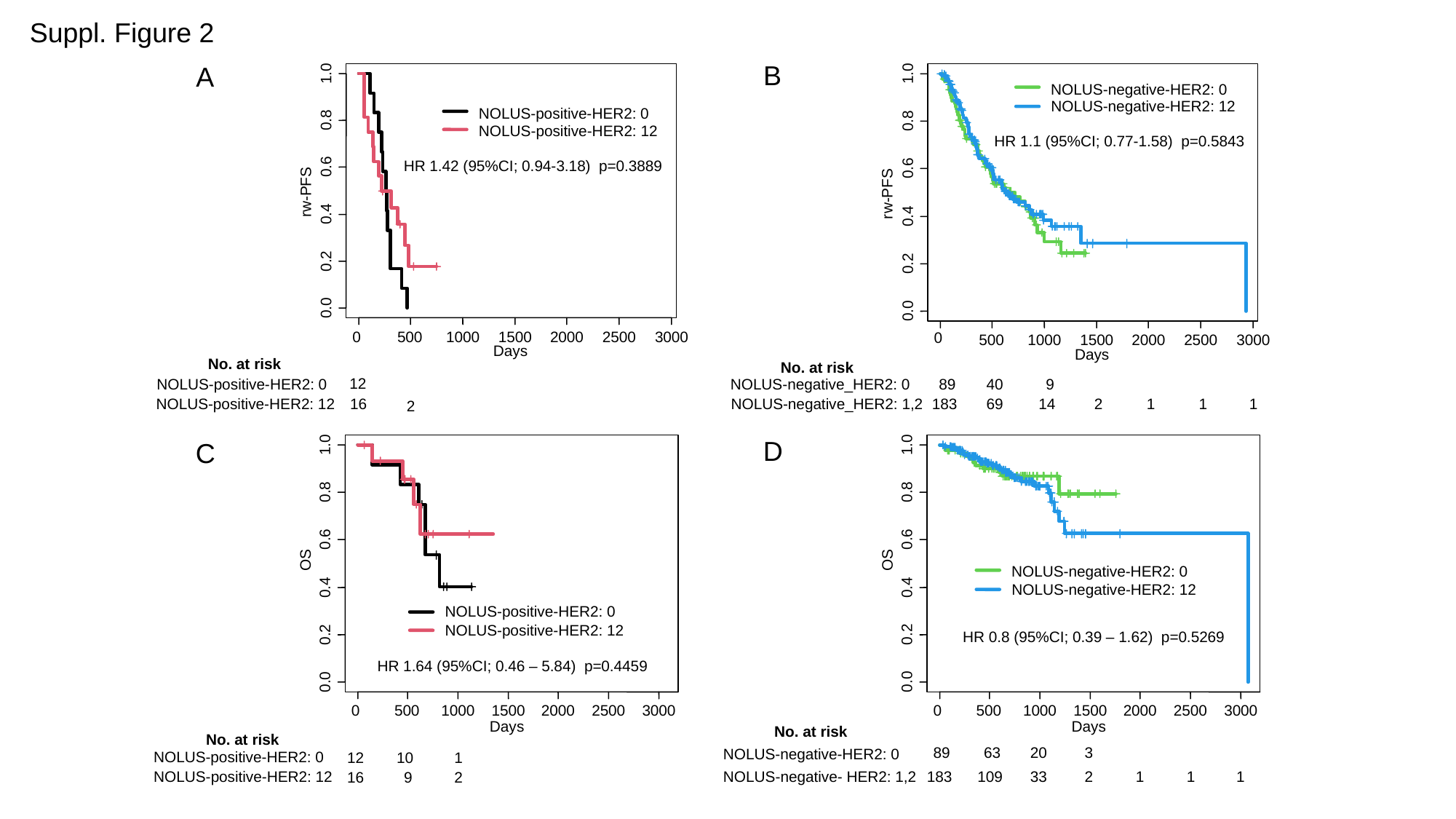

Suppl. Figure 2
B
A
1.0
NOLUS-positive-HER2: 0
NOLUS-positive-HER2: 12
0.8
HR 1.42 (95%CI; 0.94-3.18) p=0.3889
0.6
rw-PFS
0.4
0.2
0.0
0
500
1000
1500
2000
2500
3000
Days
1.0
NOLUS-negative-HER2: 0
NOLUS-negative-HER2: 12
0.8
HR 1.1 (95%CI; 0.77-1.58) p=0.5843
0.6
rw-PFS
0.4
0.2
0.0
0
500
1000
1500
2000
2500
3000
Days
No. at risk
12
NOLUS-positive-HER2: 0
NOLUS-positive-HER2: 12
16
2
No. at risk
NOLUS-negative_HER2: 0
89
40
9
NOLUS-negative_HER2: 1,2
183
69
14
2
1
1
1
D
C
1.0
0.8
0.6
OS
NOLUS-negative-HER2: 0
NOLUS-negative-HER2: 12
0.4
HR 0.8 (95%CI; 0.39 – 1.62) p=0.5269
0.2
0.0
0
500
1000
1500
2000
2500
3000
Days
1.0
0.8
0.6
OS
0.4
NOLUS-positive-HER2: 0
NOLUS-positive-HER2: 12
0.2
HR 1.64 (95%CI; 0.46 – 5.84) p=0.4459
0.0
0
500
1000
1500
2000
2500
3000
Days
No. at risk
No. at risk
NOLUS-positive-HER2: 0
12
10
1
NOLUS-positive-HER2: 12
9
2
16
89
63
20
3
NOLUS-negative-HER2: 0
NOLUS-negative- HER2: 1,2
109
33
2
1
1
1
183
